# Determining the Mode, Frequency, and Azimuthal Wave Number of ULF Waves During a HSS and Moderate Geomagnetic Storm

**DOI:** 10.1029/2017JA024877

**Published:** 2018-08-18

**Authors:** Kyle R. Murphy, Andrew R. Inglis, David G. Sibeck, I. Jonathan Rae, Clare E. J. Watt, Marcos Silveira, Ferdinand Plaschke, Seth G. Claudepierre, Rumi Nakamura

**Affiliations:** ^1^ Department of Astronomy University of Maryland College Park MD USA; ^2^ Physics Department Catholic University of America Washington DC USA; ^3^ NASA Goddard Space Flight Center, Space Weather Laboratory (674) Greenbelt MD USA; ^4^ Department of Space and Climate Physics, Mullard Space Science Laboratory University College London London UK; ^5^ Department of Meteorology University of Reading Reading UK; ^6^ Universities Space Research Association Columbia MD USA; ^7^ Space Research Institute Austrian Academy of Sciences Graz Austria; ^8^ Space Sciences Department The Aerospace Corporation El Segundo CA USA

**Keywords:** ULF waves, azimuthal wave number, mode structure, geomagnetic storms, radiation belts

## Abstract

Ultralow frequency (ULF) waves play a fundamental role in the dynamics of the inner magnetosphere and outer radiation belt during geomagnetic storms. Broadband ULF wave power can transport energetic electrons via radial diffusion, and discrete ULF wave power can energize electrons through a resonant interaction. Using observations from the Magnetospheric Multiscale mission, we characterize the evolution of ULF waves during a high‐speed solar wind stream (HSS) and moderate geomagnetic storm while there is an enhancement of the outer radiation belt. The Automated Flare Inference of Oscillations code is used to distinguish discrete ULF wave power from broadband wave power during the HSS. During periods of discrete wave power and utilizing the close separation of the Magnetospheric Multiscale spacecraft, we estimate the toroidal mode ULF azimuthal wave number throughout the geomagnetic storm. We concentrate on the toroidal mode as the HSS compresses the dayside magnetosphere resulting in an asymmetric magnetic field topology where toroidal mode waves can interact with energetic electrons. Analysis of the mode structure and wave numbers demonstrates that the generation of the observed ULF waves is a combination of externally driven waves, via the Kelvin‐Helmholtz instability, and internally driven waves, via unstable ion distributions. Further analysis of the periods and toroidal azimuthal wave numbers suggests that these waves can couple with the core electron radiation belt population via the drift resonance during the storm. The azimuthal wave number and structure of ULF wave power (broadband or discrete) have important implications for the inner magnetospheric and radiation belt dynamics.

## Key Points

- ULF wave power is systematically characterized as discrete or broadband, and the azimuthal wave number of discrete ULF waves is determined
- Storm time discrete ULF waves can couple with the core radiation belt electron population via the drift resonance
- Storm time ULF waves are the result of both external (Kelvin‐Helmholtz) and internal (plasma instabilities) generation mechanisms

## 1 Introduction

Solar wind‐magnetosphere coupling drives space weather in the Earth's magnetosphere, enabling energy to transfer from the solar wind to the magnetosphere. This coupling can occur directly via magnetic reconnection (e.g., Dungey, 1961), the Kelvin‐Helmholtz (KH) instability (e.g., Southwood, 1968), or direct buffeting of the magnetopause from solar wind pressure pulses (e.g., Kepko et al., 2002). This coupling and transfer of energy can also lead to magnetospheric activity in the form of wave‐particle interactions and resonances (e.g., Southwood et al., 1969), local plasma instabilities (e.g., Kalmoni et al., 2017; Roux et al., 1991), and the formation of a near‐Earth neutral line (Baker et al., 1996). Solar wind‐magnetospheric coupling and associated energy transfer is fundamental to the development of geomagnetic storms, the generation of Ultralow Frequency (ULF) waves, and the dynamics of the Earth's radiation belts.

Geomagnetic storms typically develop during *geoeffective* high‐speed solar wind streams (HSSs), coronal mass ejections, and corotating interaction regions (CIRs) propagating from the solar surface (e.g., Denton et al., 2006; Kataoka & Miyoshi, 2006). The solar wind‐magnetosphere coupling occurring during geomagnetic storms drives intense electromagnetic and plasma activity within a dynamic magnetosphere. For instance, the flux of energetic electrons (100s of keV to several MeV) within the Earth's outer radiation belt (2.5 < *L* < 7 R_E_) can vary throughout the belt by over 4 orders of magnitude (e.g., Degeling et al., 2013; Mann et al., 2016) and wave power in various wave modes such as Very Low Frequency chorus (e.g., Aryan et al., 2016), electromagnetic ion cyclotron Pc1 ULF waves (e.g., Halford et al., 2010), and Pc4–5 ULF (e.g., Murphy et al., 2016) waves increases exponentially. The physical processes controlling electron dynamics during geomagnetic storms and their relative contributions to electron loss and acceleration are a contentious (e.g., Friedel et al., 2002), and poorly understood (e.g., Mauk et al., 2013) topic within radiation belt research. However, in general, it is widely accepted that ULF waves can play an important role in both the loss (e.g., Brito et al., 2015; Loto'aniu et al., 2010; Mann et al., 2012; Murphy et al., 2015; Rae et al., 2018; Shprits et al., 2006; Turner et al., 2012) and energization (e.g., Brautigam & Albert, 2000; Brautigam et al., 2005; Mann et al., 2016; Ozeke et al., 2012; Ozeke, Mann, Turner, et al., 2014) of radiation belt electrons during geomagnetic storms.

Enhanced ULF wave power can be driven by high‐speed solar wind (e.g., Rae et al., 2012) via the KH instability (e.g., Claudepierre et al., 2008; Rae et al., 2005), magnetopause compressions (e.g., Murphy et al., 2016), or unstable particle distributions (e.g., Ozeke & Mann, 2008) typically observed during geomagnetic storms. These ULF waves can couple to the local charged particles through wave‐particle interactions, leading to the acceleration or loss of electrons within the inner magnetosphere (e.g., Mann et al., 2016; Ozeke, Mann, Turner, et al., 2014; Shprits et al., 2006; Turner et al., 2012). This coupling and the energization of electrons can occur via discrete phase coherent resonances such as the drift resonance (e.g., Claudepierre et al., 2013) or stochastically through ULF wave radial diffusion (e.g., Schulz & Lanzerotti, 1974). The physical mechanism of this coupling is dependent on the mode structure, azimuthal wave number *m*, and discrete or broadband nature of the ULF waves.

Drift resonance can lead to the breaking of the third adiabatic invariant, related to the azimuthal drift motion of electrons in the inner magnetosphere. In the drift resonance, discrete (monochromatic) poloidal (azimuthal electric field ***E***_*ϕ*_ and radial magnetic field ***B***_r_) or toroidal (***E***_r_ and ***B***_*ϕ*_) mode ULF waves can efficiently accelerate electrons whose frequency satisfies the resonance condition (Elkington et al., 2003; Ozeke et al., 2012). In radial diffusion, acceleration of electrons is still the result of the drift resonance; however, the process is stochastic, the result of broadband ULF wave power, such as the superposition of many monochromatic waves, or the superposition of electric field impulses (Schulz & Lanzerotti, 1974), as opposed to a single monochromatic ULF wave. Since ULF wave radial diffusion is a stochastic process it is thought to be less efficient than acceleration via discrete poloidal and toroidal ULF wave modes (e.g., Degeling et al., 2008; Elkington et al., 2003). However, one thing in common is, both the drift resonance and radial diffusion are highly dependent on the sign and value of the azimuthal wave number (e.g., Elkington et al., 2003). In the outer radiation belt clear evidence exists for both discrete resonances (e.g., Claudepierre et al., 2013; Mann et al., 2013; Zong et al., 2007) and ULF wave radial diffusion (e.g., Ozeke, Mann, Turner, et al., 2014) as mechanisms of electron acceleration and loss in the inner magnetosphere.

In radiation belt models, when assessing the relative contribution of ULF waves to electron dynamics, ULF wave power is typically assumed to be broadband (e.g., Ali et al., 2016; Brautigam & Albert, 2000; Brautigam et al., 2005; Ozeke et al., 2012) and the value of *m* is assumed to be both positive and low for all frequencies. This is in despite of evidence for discrete wave power (e.g., Claudepierre et al., 2013; Mann et al., 2013; Rae et al., 2005), and both high (e.g., Le et al., 2017) and low (e.g., Sarris et al., 2013), and positive (Sarris et al., 2013) and negative (Le et al., 2017) azimuthal wave numbers within the outer radiation belt. In general, these assumptions are made for three fundamental reasons. One, radiation belt models are based on a diffusion formalism which relies on statistical models of ULF wave power. Two, systematically determining the azimuthal wave number *m* of ULF waves is remarkably difficult; developing an understanding of how *m* varies within the magnetosphere and during differing solar wind and geomagnetic conditions and the consequences of this variation for radiation belt dynamics has, so far, proven impossible. Finally, separating discrete ULF wave power from broadband wave power is challenging and so the relative importance of resonant versus stochastic ULF wave interactions is unknown.

With the launch of the Magnetospheric Multiscale (MMS) mission (Burch et al., 2016) and the Van Allen Probes (Mauk et al., 2013), it is possible to develop a more complete understanding of how ULF waves couple to inner magnetosphere electrons either via discrete resonances and ULF wave power or via radial diffusion and broadband wave power. In this paper, we study the evolution of ULF wave power, mode, and toroidal azimuthal wave number during a moderate HSS‐driven geomagnetic storm that occurred between 24 September 2016 and 10 October 2016. In this study we concentrate on toroidal mode ULF waves as the HSS compresses the dayside magnetosphere leading to a asymmetric magnetic field topology where toroidal mode waves can resonantly couple to radiation belt electrons (e.g., Elkington et al., 2003) and introduce a new approach to determine the extent of discrete ULF wave power at MMS using the Automated Flare Inference of Oscillations (AFINO) technique originally developed for solar flare analysis (Inglis et al., 2015, 2016). We demonstrate how the azimuthal wave number *m* of discrete ULF waves may be calculated as a function of time and position in the magnetosphere using results from AFINO on the closely separated MMS spacecraft. Analysis of these azimuthal wave number allows the driver of ULF waves to be determined. Finally, we demonstrate that during this geomagnetic storm a wide range of electron energies can couple to discrete toroidal mode ULF waves with the appropriate azimuthal wave numbers; this coupling potentially contributing to an efficient energization of radiation belt electrons as observed by the Van Allen Probes.

## 2 Data and Methodology

### 2.1 Data

The MMS mission was launched on 13 March 2015 and consists of four identically instrumented spacecraft flying in a tight tetrahedral formation. MMS was designed to study the microphysics of reconnection both along the magnetopause at ~10–12 R_E_ and in the magnetotail at the near‐Earth neutral line ~25 R_E_ (Burch et al., 2016). Though the main scientific objective of MMS is to improve our understanding of reconnection, the unique timing of the launch of MMS and its orbital configuration are ideal for studying the dynamics of ULF waves. MMS was launched at the end of solar maximum and beginning of the declining phase of solar cycle 24. During this period of the solar cycle the occurrence rate increases for both HSS and CIRs which efficiently drive ULF wave power in the magnetosphere (e.g., Rae et al., 2012). Further, the close separation of MMS is ideal to characterize the azimuthal wave number of ULF waves.

**Figure 1:**
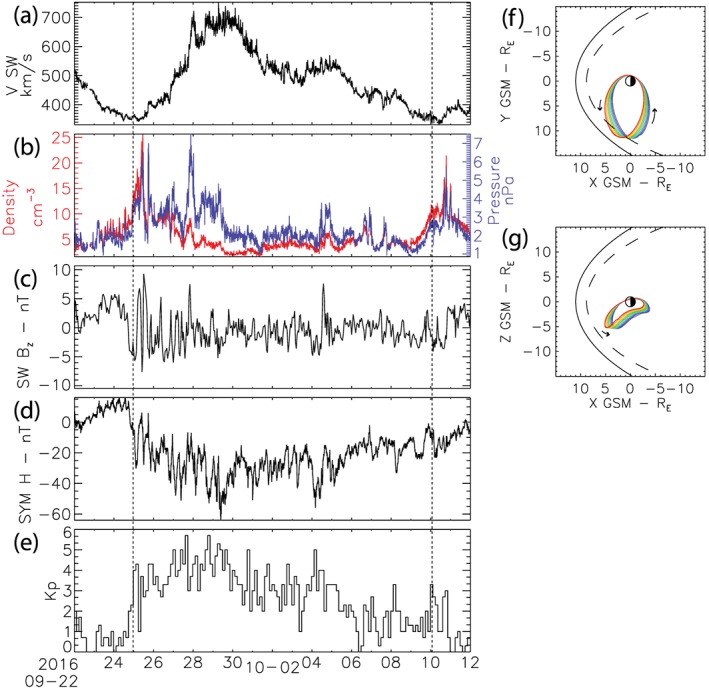
The high‐speed solar wind stream between 25 September 2016 and 10 October 2016. From top to bottom, Figure 1 shows (a) the solar wind speed V SW, (b) the solar wind density (red) and pressure (blue), (c) the GSM B_z_ component of the magnetic field in the solar wind, (d) the geomagnetic Sym‐H index, and (e) the Kp geomagnetic index. Panels (f) and (g) illustrate the elliptical orbit of MMS relative to the Earth during this time interval starting at blue at the beginning of the interval and transitioning to red toward the end of the interval. The solid and dashed lines in panels (f) and (g) show the typical location of the magnetopause and the location of the magnetopause during fast solar wind.

In this study we use Flux Gate Magnetometer (FGM) data (Russell et al., 2016) to characterize the evolving properties of ULF waves during a HSS observed between 25 September and 10 October 2016 (Figure 1). The FGM measures the three‐component DC magnetic field; in the fast and slow survey modes, utilized here, the DC magnetic field is measured with a 16‐ and 8‐Hz cadence (Russell et al., 2016), respectively. The configuration of the MMS tetrahedron and the high‐cadence of the FGM are ideal for determining the azimuthal wave number *m* of ULF waves (e.g., Le et al., 2017). However, calculating *m* requires the robust identification of discrete ULF wave power. In this study we use the AFINO (described in section 2.2) code (Inglis et al., 2015, 2016) to identify discrete ULF wave power observed by MMS and implement a new algorithm utilizing the cross‐phase technique (described in section 2.3) to calculate *m* throughout the HSS and moderate geomagnetic storm.

For the purposes of this study we are interested in ULF wave oscillations between ~0.5 and ~25 mHz (~40–~2,000 s) spanning the Pc 4–5 wave band (45–600 s) which can interact with outer radiation belt electrons (e.g., Ozeke et al., 2012). The magnetic field data are rotated into a nominal field‐aligned coordinate system using the same methodology outlined in Le et al. (2017). In this field‐aligned coordinate system, ***B***_∥_ is the compressional component of the magnetic field and lies along the background field, ***B***_*ϕ*_ is the toroidal magnetic field and is perpendicular to ***B***_∥_ and the satellite radial position vector, and ***B***_r_ is the poloidal magnetic field and completes the right‐handed coordinate system. It is important to note that MMS probes have a spin rate of ~20 s leading to a clear spin tone oscillation at 50 mHz just outside of the Pc 4–5 frequency range. To ensure that the MMS spin tone does not contaminate the Pc 4–5 wave power and subsequent identification of discrete waves, the data are down sampled to 15 s per sample using a linear interpolation then low‐pass filtered at twice the Nyquist frequency (33.3 mHz) to remove aliasing of frequencies above the Nyquist frequency from the original time series in the decimated time series. This removes the spin tone while preserving the Pc 4–5 wave power. When identifying discrete wave power using AFINO the 15‐s data are used; however, when calculating the azimuthal wave number *m*, the higher‐frequency data are used as the high sampling rate is necessary for calculating the phase difference of ULF waves between the closely separated MMS probes (described below).

Figure 1 shows the solar wind and geomagnetic conditions during the interval of interest from 25 September to 10 October 2016, marked by dashed lines. The solar wind and geomagnetic indices are from the OMNIweb space physics data facility; the solar wind data have been time shifted to the nominal location of the Earth's bow shock (King & Papitashvili, 2005). The HSS is characterized by an increase in solar wind density and dynamic pressure (Figure 1b) at the front of the HSS followed by an interval of high‐speed solar wind (>500 km/s) lasting for nearly 10 days (Figure 1a; e.g., Denton & Borovsky, 2002). For the duration of the interval the southward component of the interplanetary magnetic field (IMF) is predominantly negative albeit with clear oscillations that are typically associated with HSSs and CIRs (Kataoka & Miyoshi, 2006). Figures 1d and 1e show the geomagnetic indices Sym‐H and Kp during the interval, respectively. The HSS leads to a moderate geomagnetic storm with a minimum Sym‐H just below −60 nT and a peak Kp of 6−. During the HSS MMS is located on the duskside flank (Figures 1f and 1g); the solid and dashed lines show the typical location of the magnetopause and the location of the magnetopause during fast solar wind (Sibeck et al., 1991). The HSS solar wind stream coupled with the location of MMS on the duskside flank provides an ideal opportunity to investigate the evolution of discrete and broadband ULF wave power and azimuthal wave number throughout a geomagnetic storm.

**Figure 2:**
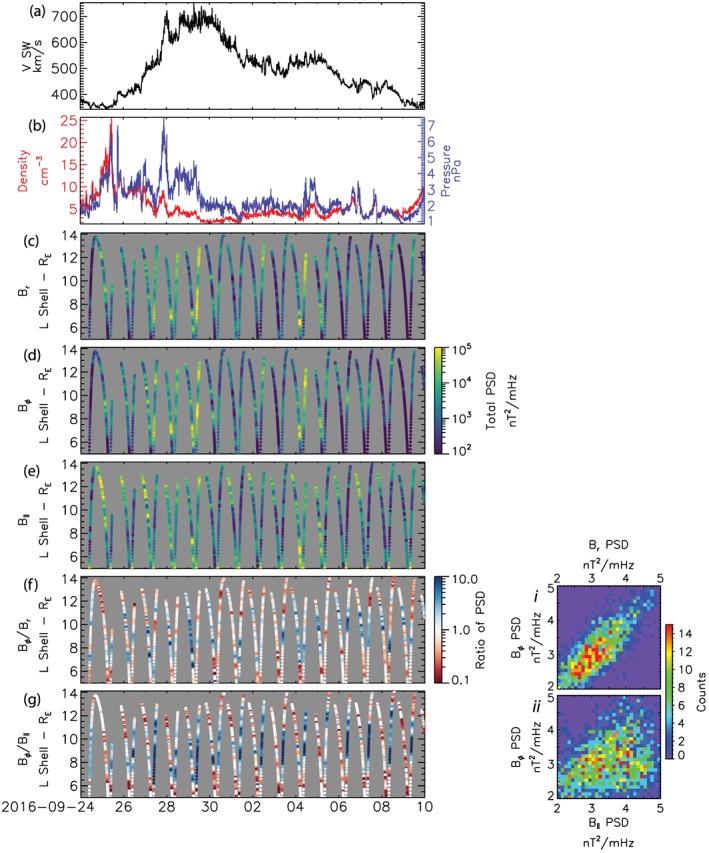
Solar wind (a and b) and ULF wave (c–g) dynamics during the HSS. (a) Solar wind speed V SW. (b) Solar wind density (red) and dynamic pressure (blue). (c–e) Total ULF wave poloidal, toroidal, and compressional wave power through the HSS. (f and g) Ratio of toroidal wave power to poloidal and compressional wave power, respectively. (i and ii) Two‐dimensional histograms showing the relation between ULF wave power in the three different wave modes throughout the HSS allowing the dominant ULF wave mode to be assessed. (i) Poloidal (panel c) versus toroidal (panel d) wave power. (ii) Compressional (panel e) versus toroidal wave power (panel d).

Figure 2 shows the evolution of the solar wind and ULF wave power through the HSS. The top two panels of Figure 2 show the solar wind speed (a) and density and pressure (red and blue, respectively) for comparison to the ULF wave power observed through the HSS shown in Figures 2c–2g. The ULF wave power is calculated for MMS‐1 from 30‐min windows using the Fast Fourier Transform. The power is then summed between the Pc4–5 ranges (30–1,500 s) to provide an estimate of total ULF wave power. The 30‐min windows are then slid every 5 min giving ULF wave power as function of L shell (radial distance) throughout the HSS, in the same manner as a dynamic power spectra. Periods when MMS is outside the magnetosphere (e.g., 25 September 2019) are ignored in this analysis. Note that throughout the HSS the interaction of the solar wind with the magnetosphere causes the magnetopause to move inward compressing the dayside magnetosphere. On the dusk flank MMS observes the magnetopause as close in as 10 R_E_, corresponding to about 7 R_E_ at the nose of the magnetopause (Shue et al., 1998).

During the HSS ULF wave power (Figures 2c–2e) is largest, 10^5^ nT^2^/mHz, during the beginning of the HSS when the solar wind speed, density, and dynamic pressure peak. Following the peak in solar wind speed ULF wave power decays (10^3^–10^4^ nT^2^/mHz), though there are clear increases in wave power to values >10^4^ nT^2^/mHz associated with density and pressure peaks throughout the event, for example, 4–5 October 2016. The bottom two panels of Figure 2 show the ratio between the toroidal and poloidal and the compressional and poloidal wave power during the HSS. The insets of Figure 2 show the 2‐D histogram of toroidal wave power versus poloidal (i) and compressional (ii) wave power. The ratios allow the dominant mode of ULF wave power to be diagnosed. Throughout the HSS compressional power tends to peak and dominate at high L (*L* > 10) near the magnetopause and deep in the inner magnetosphere where the ratios are >1 (*L* < 6, Figures 2e and 2f). Toroidal and poloidal wave power tend to peak in the middle to inner magnetosphere (*L* 7–10) with enhanced power between 10^4^–10^5^ nT^2^/mHz (Figures 2c and 2d). Comparing Figures 2f and 2g, we see that poloidal and toroidal wave power tend to be of similar magnitudes with ratios ~1 (low values in the ratios of Figure 2f) and in general dominate the wave power in the inner magnetosphere where the ratio between toroidal and compressional power is >1 (dark blue in Figure 2g). This is confirmed by the insets; the 2‐D histogram of poloidal wave power versus toroidal wave power (Inset i) shows a clear linear relation demonstrating that the toroidal and poloidal wave power are of similar magnitudes. Comparing toroidal and compressional wave power (Inset ii), the relation is less clear, the result of compressional power dominating near the magnetopause and at low *L* values.

Poloidal and toroidal wave power are key to ULF wave energization of radiation belt electrons (Elkington et al., 1999, 2003). The accumulation of both poloidal and toroidal wave power in the magnetosphere suggests that ULF waves during this HSS may be a key factor in the energization of radiation belt electrons. In subsequent sections we introduce two new methods to systematically identify discrete ULF waves and calculate azimuthal wave numbers and discuss the implications of discrete wave power during the HSS for radiation belt and energetic electron dynamics.

### 2.2 The AFINO Code

To robustly analyze the magnetospheric data obtained by MMS during this HSS, we utilize the AFINO code (Inglis et al., 2015, 2016). AFINO was originally developed as a tool to statistically investigate the occurrence of quasiperiodic pulsations in a large sample of solar flares, as such studies cannot be achieved via manual means. However, AFINO is designed to systematically identify discrete power and may be adapted to operate on a variety of time series, including solar wind and magnetospheric data, making it ideal for studying discrete ULF waves with MMS. For a given time series, AFINO operates by examining the Fourier power spectrum of the series and uses a model comparison approach to find the best representation of the data. In this way we can quantitatively and systematically identify intervals within a time series where discrete power is present. Here we summarize the key steps in the method.

The first step is to apodize the input data by normalizing by the mean and applying a Hanning window to the input time series (Inglis et al., 2016)—in this case the magnetic field measured by the MMS FGM. The normalization of the signal is for convenience only; however, the application of a window function is necessary to mitigate the effects of the finite‐duration time series on the Fourier power spectrum. The next stage, and the key element of the AFINO procedure, is to perform a model comparison on the Fourier power spectrum of the time series. AFINO is flexible regarding both the choice of models describing the relation between frequency and power, and the range of data being included in the fitting procedure. In the original flare‐based studies, AFINO was implemented using a number of functional forms for the Fourier power spectra, including a power law, a broken power law, and a power law plus Gaussian enhancement (Inglis et al., 2015, 2016). The latter model is designed to represent a power spectrum containing discrete power, while the other models provide alternative null hypotheses. In this current work, we consider two models for describing the Fourier power spectrum of solar wind magnetometer data: a single power law plus constant model (*S*_0_), which represents broadband power, and a power law plus constant model with an additional localized enhancement (*S*_1_). This second model represents a discrete power signature.

The choice of the simplest model *S*_0_ is based on the observation that power law Fourier power spectra are a common property of many astrophysical, solar, and magnetospheric phenomena (Cenko et al., 2010; Gruber et al., 2011; Huppenkothen et al., 2013; Inglis et al., 2015; McHardy et al., 2006; Murphy et al., 2011; Rae et al., 2012) and that such power laws can lead naturally to the appearance of bursty features in time series. This power law must be accounted for in models to avoid a drastic overestimation of the significance of localized peaks in the power spectrum (Gruber et al., 2011; Vaughan, 2005). The second model *S*_1_ is equivalent to model *S*_0_ plus an extra term corresponding to a Gaussian enhancement in log frequency space. This model component is designed to represent discrete power, that is, excess power in a localized frequency range. Formally, these models may be written as

(1) $$S_{0}\left(f\right)=A_{0}f^{-\alpha_{0}}+C_{0}$$

(2) $$S_{1}\left(f\right)=A_{1}f^{-\alpha_{1}}+B_{1}\exp\left(\frac{-{\left(\ln\left(f\right)-\ln\left(f_{p}\right)\right)}^{2}}{2\sigma^{2}}\right)+C_{1}\;$$

where *S*_*x*_ is the two models, *f* is frequency, *α*_*x*_ is the power law exponent in each model, and *f*_*p*_ and *σ* are the frequency and width of the Gaussian enhancement in model 1, respectively. In equations (1) and (2) *A*_*x*_, *B*_*x*_, and *C*_*x*_ are constants defining the slope of the power law, the amplitude of the Gaussian enhancement and the intercept of power law, respectively.

In order to fit each model to the Fourier power spectrum, we determine the maximum likelihood *L* for each model with respect to the data. For Fourier power spectra, the uncertainty in the data points is exponentially distributed (e.g., Vaughan, 2005, 2010). Hence, the likelihood function may be written as

(3) $$L=\prod_{j=1}^{N/2}\frac{1}{s_{j}}\exp\left(-\frac{i_{j}}{s_{j}}\right)\;$$

where *I* = (*i*_1_, …, *i*_*N*/2_) represent the observed Fourier power at frequency *f*_*j*_ for a time series of length *N* and *S* = (*s*_1_, …, *s*_*N*/2_) represents the model of the Fourier power spectra. In this work, the maximum likelihood (or equivalently the minimum negative log likelihood) is determined using fitting tools provided by SciPy (Jones et al., 2001). To ensure that the true maximum likelihood is found, each fit is initialized multiple times with randomized initial guess parameters, and the fit that best maximizes the likelihood across all iterations is used.

Once the fitting of each model is completed, AFINO performs a model comparison test using the Bayesian Information Criterion (BIC) to determine which model is more appropriate. The BIC is closely related to the maximum likelihood *L*, and the *BIC* comparison test functions similarly to a likelihood ratio test. The BIC (for large *N*) is given by (Burnham & Anderson, 2004)

(4) $$\mathrm{BIC}=-2\ln\left(L\right)+k\ln\left(N/2\right)\;$$

where *L* is the maximum likelihood described above, *k* is the number of free parameters and *N* is the number of data points in the power spectrum. The key concept of BIC is that there is a built‐in penalty for adding complexity to the model. In this work, this is particularly useful because models *S*_0_ and *S*_1_ are nested. Hence, since *S*_1_ contains more free parameters it should always better maximize the likelihood—or equivalently minimize the negative log likelihood—than model *S*_0_. Using the *BIC* value to compare models therefore tests whether the added complexity offered by model *S*_1_ is sufficiently justified. For the purpose of our study, periods of discrete ULF wave power are identified when ΔBIC *=* BIC_0_ − BIC_1_ > 10. Whenever BIC_1_ *<* BIC_0_ the discrete power model *S*_1_ is preferred to the single power law model *S*_0_, however applying the more stringent constraint that ΔBIC > 10 ensures that we identify time periods where the discrete power model is strongly preferred (e.g., Burnham & Anderson, 2004).

A limitation to the above approach is that neither likelihood nor BIC determines whether the tested models are good choices in absolute terms—all of the chosen models could be poor representations of the observed data. Hence, in order to provide a secondary check and determine if models *S*_0_ and *S*_1_ are consistent with the data we utilize the χ^2^‐like statistic for exponentially distributed data described by Nita et al. (2014), who showed that an appropriate statistic may be written as

(5) $$\chi_{\upsilon }^{2}=\frac{1}{\upsilon }\sum_{j-1}^{n}{\left(1-\rho_{j}\right)}^{2}\;$$

for a single Fourier power spectral sample, where *ρ*_*j*_ *= i*_*j*_ */s*_*j*_ is referred to as the sample‐to‐model estimator, that is, the ratio of the data to the best fit model (see Nita et al., 2014, equation (5)), and *ν* is the number of degrees of freedom. We determine χ^2^_υ_ for each model‐data pair using the approximate expression for the probability density function of χ^2^_υ_ (Nita et al., 2014, Appendix A) and subsequently derive a probability value *p* of the data for each model. In this work, the main purpose of this statistic is to identify intervals and events where the models are clearly inappropriate for the data. Hence, using this test, we discard from consideration events where the derived *p* value is anomalous, defined here as *p* < 0.01, regardless of the result of the BIC test. For such cases the model comparison via ΔBIC has limited value; in these cases we can say only that additional models should be tested.

**Figure 3:**
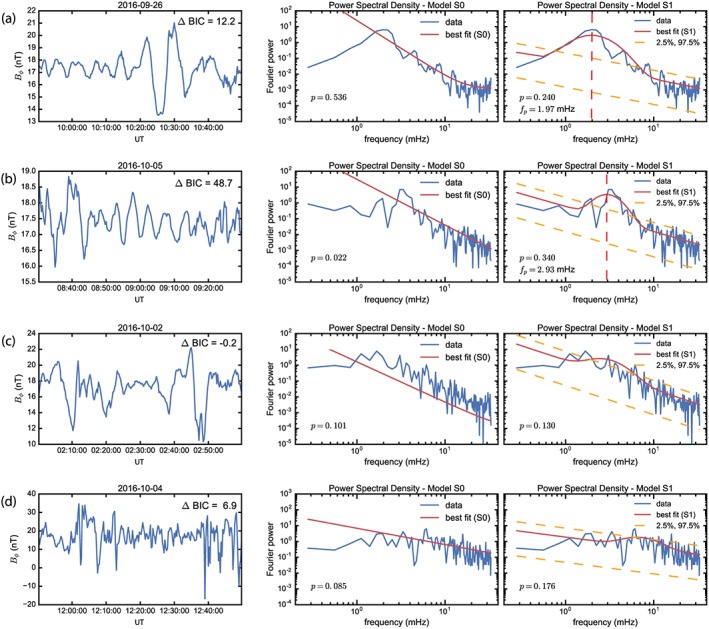
Examples of intervals in the MMS field data analyzed by AFINO. In each case, the toroidal component B_φ_ component of the magnetic field was analyzed. For each row (a)–(d) we show the analyzed input magnetic field time series (left panel), the best fit to the single power law model *S*_0_ (center), and the best fit to the discrete power law model *S*_1_ (right). Each model is fit over the entire frequency domain. The ΔBIC = BIC_0_ − BIC_1_ is shown in the top right of the first column in each row. The orange dashed lines in the right panels represent the ±2*σ* deviation from the power law component of the model fit as an indication of the significance of any peak in power. Rows (a) and (b) show intervals where strong discrete power was identified such that model *S*_1_ is a better representation of the data then *S*_0_, ΔBIC > 10. The frequency of the discrete power *f*_*p*_ (cf. equation (1)) in panels (a) and (b) is identified by the dashed red line. Rows (c) and (d) illustrate intervals of broadband power where no discrete wave power was identified, and the Fourier spectra are better characterized by *S*_0_. In each panel *p* is the *p* value and is a measure of the goodness of fit of each model. Any model fit (*S*_0_ or *S*_1_), where *p* < 0.01, are considered poor fits.

Figure 3 shows four examples of the AFINO code run on data obtained from the MMS FGM. The left column is the input time series given to AFINO, shown prior to normalization and windowing. In this study the input data is 1‐hr intervals of the toroidal magnetic field. A 1‐hr interval is used as input as it provides the necessary frequency resolution for fitting the data with the two models *S*_0_ and *S*_1_ and the subsequent model‐data comparison. The toroidal magnetic field ***B***_*ϕ*_ is used as these waves have been shown to be able to interact with electrons in an asymmetric magnetic field topology characteristic of the Earth's magnetosphere (Elkington et al., 2003). The toroidal magnetic field is also used to subsequently calculate the azimuthal wave number *m*. The middle and right columns are the data‐model comparison of the Fourier power spectra to the power law *S*_0_ and power law plus bump *S*_1_ models, respectively. Figures 3a and 3b show two periods of ULF wave activity observed at MMS during the HSS, in each interval BIC_0_ – BIC_1_ > 10 and thus are characterized as intervals of discrete power. Comparing models *S*_0_ (middle) and *S*_1_ (right) of rows (a) and (b), it is clear that the power law plus bump is a better representation of the power spectra than the power law alone during these intervals. The bottom two rows (c) and (d) of Figure 3 show intervals of broadband power where model *S*_0_ is preferred to *S*_1_. In each case both models *S*_0_ and *S*_1_ are fitted over the entire Fourier spectrum.

To apply AFINO to the MMS FGM data during the HSS shown in Figures 1 and 2, we select continuous 1‐hr intervals of the 15‐s‐filtered toroidal magnetic field ***B***_*ϕ*_ data of MMS‐1 described in section 2.1. This choice of interval length provides sufficient Fourier resolution and data points to ensure that the model comparison described above operate effectively to study wave power in the Pc 4–5 range. However, as a result of windowing the signals input into AFINO, features that occur near the beginning or the end of the time window may be suppressed. To account for this, we adopt a sliding window approach, whereby we continuously slide the 1‐hr interval forward in 10‐min increments. Using overlapping windows ensures that transient, short‐lived features in the magnetometer data will not be missed by the AFINO algorithm. For the purpose of this study we also restrict the location of the bump *f*_*p*_ in model *S*_1_ to 1–40 mHz (25–1,000 s), the Pc4–5 frequency range.

### 2.3 Azimuthal Wave Number

The ULF azimuthal wave number is key to any assessment of the contribution of ULF wave‐particle interactions to radiation belt dynamics. Typically, the azimuthal wave number *m* of a ULF wave can be calculated by determining the phase difference between two‐point observations at the same latitude or L shell but which are separated in azimuth (e.g., Chisham & Mann, 1999). Historically, *m* has been calculated using ground‐based observations which provide the necessary multipoint observations to determine the phase shift as a function of azimuthal separation (e.g., Rae et al., 2005; Yeoman et al., 2012). With an increasing number of high‐fidelity observations in the magnetosphere and multipoint missions, *m* can now be calculated in situ using magnetic field observations from closely spaced observations such as Geostationary Operational Environmental Satellite system (Rae et al., 2005), Time History of Events and Macroscale Interactions during Substorms (Sarris et al., 2013), or MMS (Le et al., 2017) or from particle observations with sufficient energy resolution and pitch angle resolution and energy range (Claudepierre et al., 2013; Min et al., 2017; Ren et al., 2017; Zhou et al., 2015). Here we use the high‐resolution magnetic field observations and multipoint observations from MMS and the results from AFINO to systematically calculate *m* by analyzing the phase difference between pairs of MMS observations during periods of discrete ULF wave power.

The phase difference between two MMS observations can be calculated using the Fourier spectra, cross spectra, and cross phase of the two time series (e.g., Waters et al., 1991). The cross phase of two time series provides an estimation of the phase difference between the two series as a function of frequency (or period); however, the cross phase at any frequency is only valid only if there is clear wave power at that frequency. Using the output from AFINO we can identify both intervals of discrete ULF wave activity and the corresponding frequency of the ULF wave in order to determine the phase difference and hence the azimuthal wave number. During these intervals model *S*_1_ is the best representation of the data, ΔBIC > 10, and the frequency of the discrete ULF wave is given by *f*_*p*_ from equation (1) and illustrated by the dashed red line in Figures 3a and 3b. For each MMS pair (six in total) the phase difference is calculated between the toroidal magnetic field ***B***_*ϕ*_ as a function of spacecraft azimuthal separation at the frequency of the wave. Linear regression between the spacecraft azimuthal separation and phase difference is then used to determine the azimuthal wave number *m*. This method has two benefits to previous methods for determining *m*. First, the multiple MMS pairs and close separation between the satellites removes the 2π ambiguity in calculating the phase difference. Accounting for the azimuthal separation of the MMS phase wrapping can be neglected for |*m*| ≲ 1,200. In the inner magnetosphere the azimuthal wave number of ULF waves is typically |*m*| < 100 (e.g., Chisham & Mann, 1999; Plaschke et al., 2008) thus phase wrapping with MMS can in general be neglected for this study. The close separation of the MMS spacecraft also allows us to assume that the four probes are on the same L shell; the variation in L shell between the spacecraft being less than 0.01 R_E_. Second, determining *m* using a linear fit to the phase difference as a function of satellite separation allows the estimation of *m* as well as an uncertainty in *m*, *δm*.

Nominally, this method works best if the wave identified by AFINO is present throughout the window over which the Fourier spectra and cross phase is calculated. However, ULF waves can be localized in both space and time; any localization of the wave signal identified by AFINO will introduce uncertainty in the estimation of the phase difference between MMS pairs. As with any spectral analysis there is a trade‐off between identifying waves localized in time and waves localized in frequency. In this case, the AFINO code requires a 1‐hr window of data to provide the necessary frequency resolution with which to clearly identify peaks at lower frequencies within the power spectra. However, to accurately calculate the cross phase between each MMS pair a shorter window is required to reduce uncertainty in the calculation of the phase difference in cases when ULF wave power is temporally localized. As a result of this trade‐off, when calculating the cross phase between MMS pairs the 1‐hr window during which discrete power was identified is separated into 30‐min segments. For each 30‐min window the cross phase is only calculated if the amplitude of the wave exceeds a specified amplitude threshold for each MMS pair. For this particular study the threshold is determined from the amplitude of the discrete wave packet calculated from the 1‐hr window from AFINO. This threshold provides the average amplitude of the wave in a 1‐hr window (e.g., Press & Vetterling, 1992). If the average amplitude of the wave in a 30‐min window exceeds that of the threshold the cross phase is calculated. The 30‐min window is then shifted 15 min to ensure that the wave is accurately identified in time, in the same manner as the AFINO algorithm described above. This provides a nominal 18 cross‐phase points (three 30‐min windows and six MMS pairs) with which to calculate *m* in a 1‐hr window.

**Figure 4:**
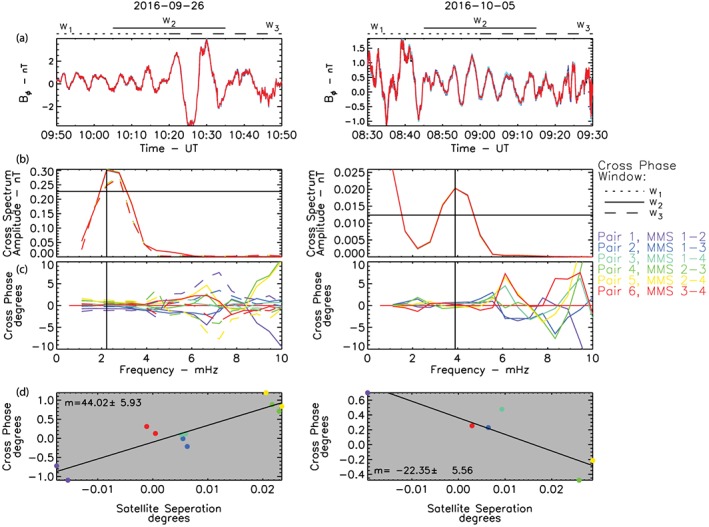
Examples of the calculation of the ULF azimuthal wave number during two periods of discrete wave power identified by the AFINO. (a) The ***B***_*ϕ*_ component of the four MMS probes, the dotted, solid, and dashed lines horizontal lines, illustrates the three windows used to calculate the cross spectra and cross phase. (b) Cross spectra and (c) cross phase of each of the MMS pairs and windows that satisfy the amplitude threshold (horizontal line) at the frequency f_p_ of the discrete wave identified by AFINO (vertical line). (d) Spacecraft azimuthal separation versus cross phase for each MMS pair, the solid line is a linear fit to the data, the slope and uncertainty in the slope provide an estimation of *m* and uncertainty *m* during the interval. Note that in panels (b)–(d) only the 30‐min windows satisfying the amplitude threshold are plotted (see text). In panels (b)–(d) the colors represent each of the six MMS satellite pairs.

**Figure 5:**
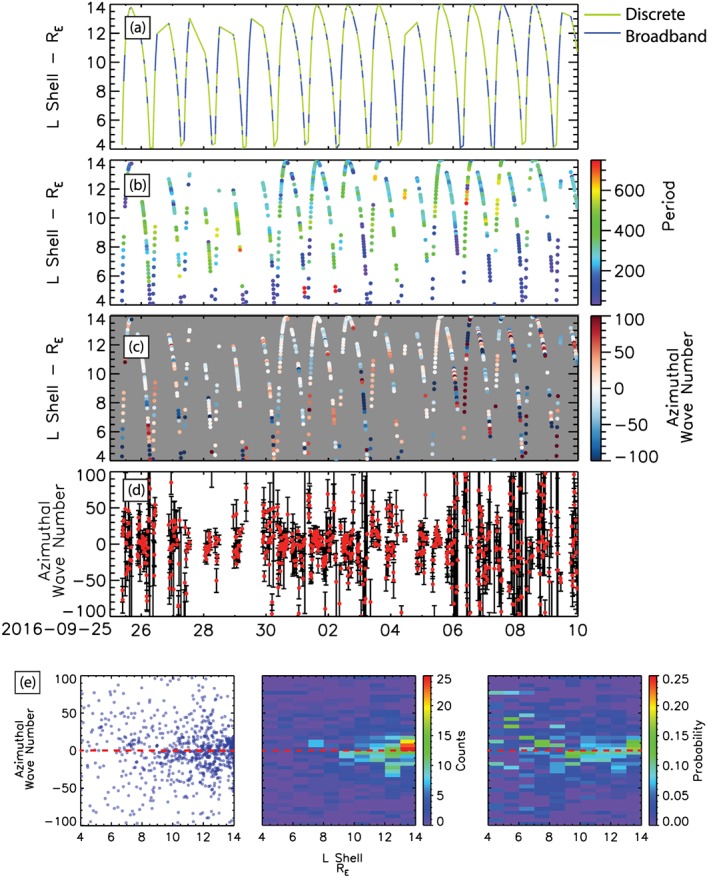
Evolution of wave power and azimuthal wave number through the high‐speed solar wind stream. (a) Distribution of discrete (green) and broadband (blue) power. (b) The period of discrete waves identified by AFINO. (c) The azimuthal wave number as a function of L during discrete wave power. (d) The azimuthal wave number *m* and uncertainty in *m*, *δm*. The L shells plotted in panels (a)–(d) are the median L shell value in the 1‐hr AFINO. (e) Distribution, 2‐D histogram, and probability density function of the azimuthal wave number *m* as function of L shell.

Figure 4 shows two examples of this methodology during two intervals of discrete power identified by AFINO (Figures 3a and 3b). The top panel shows the toroidal component of the magnetic field at each MMS probe and the three windows used to calculate the cross‐phase spectra. The close separation of the MMS satellites and the long duration of the plot (Figure 4a) makes it impossible to visually discern any phase difference between the satellites. Figure 4b shows the cross spectra between each MMS pair. The vertical line identifies the frequency of the discrete wave identified by AFINO, and the horizontal line identifies the amplitude threshold used to calculate the cross phase; if the cross power at the frequency identified by AFINO is below this threshold the cross phase for that window is not calculated. In both cases the peak in the cross spectra is coincident with the peak in discrete power identified by AFINO to within the frequency resolution of the spectra. The third panel (Figure 5c) shows the cross phase as a function of frequency for each of the MMS pair which satisfy the c. The fourth panel (Figure 5d) shows the cross phase for each MMS pair as a function of spacecraft separation. A linear fit to the data is also shown in the fourth panel (Figure 5d); the slope and uncertainty in slope provide an estimate of *m* and the uncertainty in *m*, *δm*, shown in the top left. In the first example (left) the amplitude of the ULF wave is quite large and the spread in the cross phase is small; in the second example (right) the amplitude of the wave is smaller and the spread in the cross phase is larger. As described above this is an inherent limitation in using the cross spectra to determine the cross phase between two signals. However, the linear regression and multiple MMS pairs allow the uncertainty in *m* to be estimated accounting for the spread in cross phase between MMS pairs.

## 3 Results

**Figure 6:**
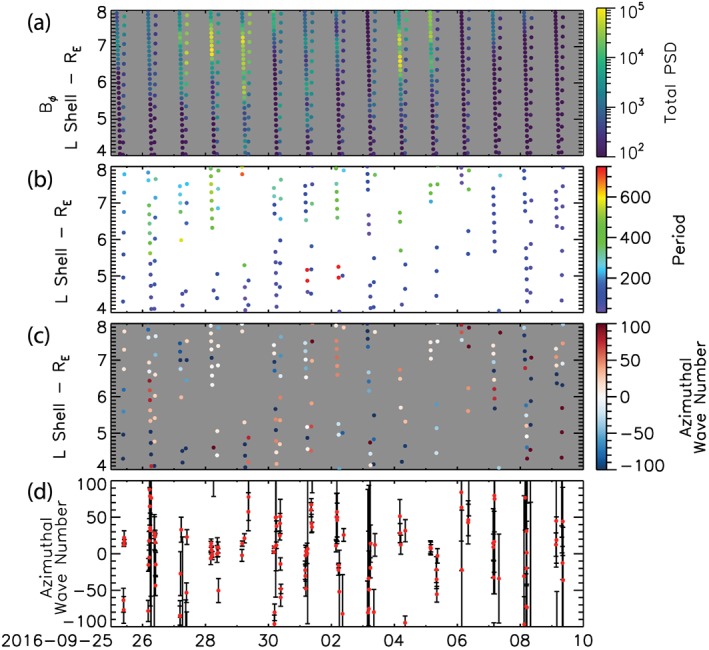
A zoom in of Figures 2 and 5 in the region of the outer radiation belt, 4 < *L* < 8 R_E_. (a) Total azimuthal wave power. (b) Period of discrete ULF wave activity. (c) Azimuthal wave number and (d) azimuthal wave number *m* and *δm*.

**Figure 7:**
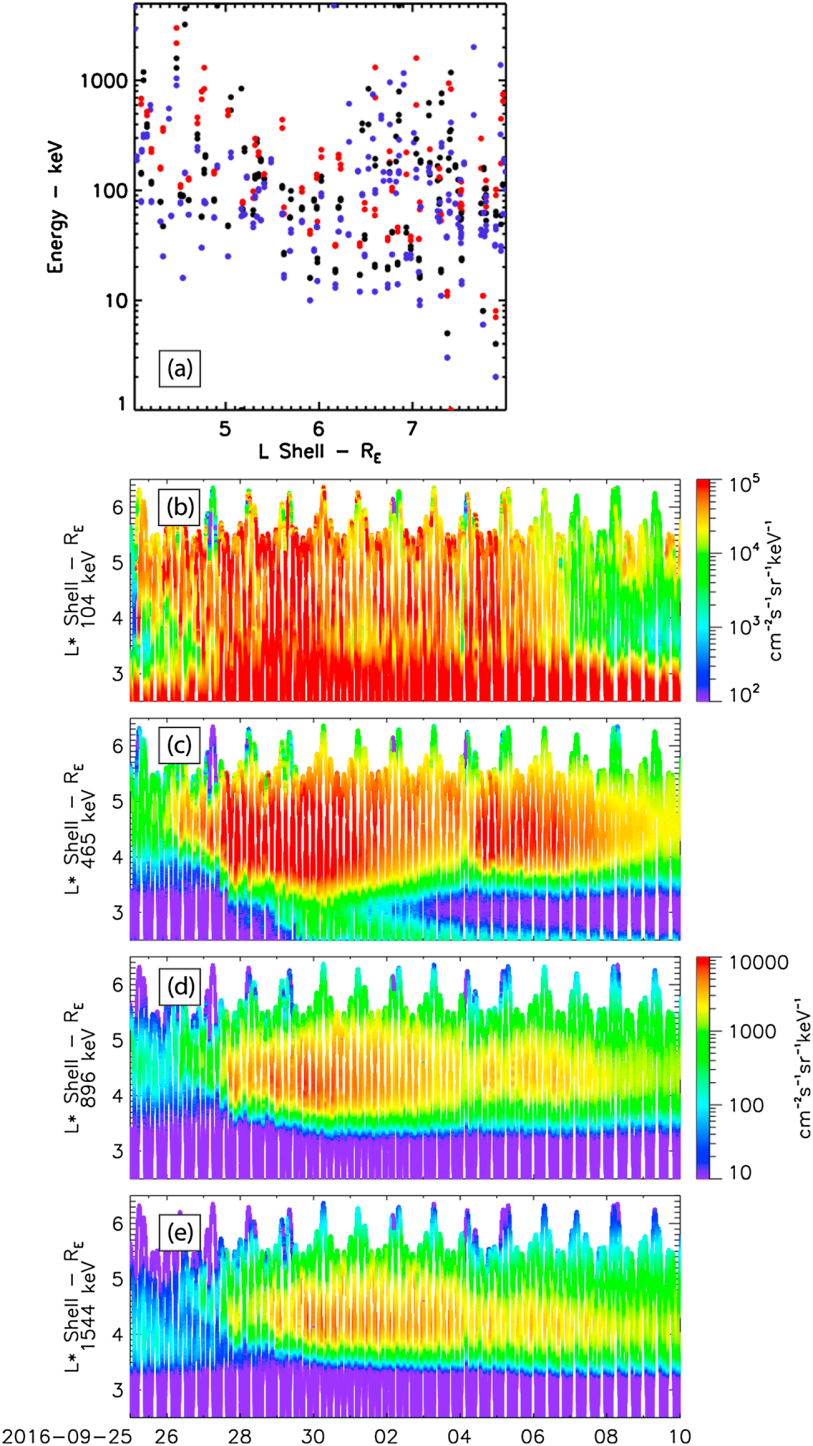
(a) The energy of electrons that can interact with the discrete ULF waves via the drift resonance throughout the outer radiation belt region is shown in Figure 6. Black points are the resonant electron energy for only positive *m* values. Red and blue points show the resonant electron energy when the uncertainties in *m* are considered and *m* remains positive (red, *m* + *δm* > 0; blue, *m* − *δm* > 0). (b–e) Equatorial electron flux (90° pitch angle) as a function of L* through the high‐speed solar wind stream. The electron energies shown correspond to the energy of electrons that can interact with discrete ultralow frequency wave power observed at MMS. Panels (c)–(e) share the same color bar plotted with (d).

Figure 5 shows results from the combined AFINO and azimuthal wave number analysis described in the previous sections during the HSS from 25 September to 10 October 2016. Figures 5a and 5b show the results of the AFINO algorithm applied to the ***B***_*ϕ*_ component of the magnetic field during the HSS described in Figures 1 and 2. Here, the results are presented as a function of both time and L shell; the L shells plotted in Figure 5 (and Figures 6 and 7) are the median L shell value in the 1‐hr AFINO. Figure 5a shows intervals of broadband (blue) and discrete power (green), and Figure 5b shows the wave period of ULF wave packets during intervals of discrete power. From this we can see that discrete power intervals are distributed throughout the HSS but most heavily concentrated in the period between 30 September 2016 and 4 October 2016 during a prolonged period of nearly steady high‐speed solar wind, *v* ~550 km/s, and during which there are small variation in the solar wind density and pressure. Figure 5b shows that the period of the discrete ULF wave power during the HSS is generally between 200 and 400 s (2.5–5 mHz) in the Pc5 range. Also, evident in Figure 5b is that shorter period waves are typically observed at lower *L* values (blue color), consistent with the period of wave resulting from the Alfven continuum within the magnetosphere. Although the perigee for the MMS spacecraft is ~1.2 R_E_, intervals where L falls below ~4 R_E_ are not analyzed by the AFINO algorithm. This is because in these regions, the changes in magnetic field are dominated by the extremely rapid motion of MMS through the local dipolar magnetosphere. These rapid changes dominate the resulting Fourier spectra and compromise the model comparison procedure. Fortunately, due to the elliptical orbit of MMS, relatively little time is spent in the low L region.

Figure 5c shows the estimate of the azimuthal wave number *m* as a function of time and L using the cross‐phase technique. As discussed in section 2.3, the wave number *m* is only estimated during intervals where discrete wave power is identified using the AFINO algorithm. From Figure 5c we can see that both positive and negative values of *m* are observed. At high L the *m* numbers are typically low *m* < 20; in the inner magnetosphere the *m* shows more variation and can be both positive and negative. Figure 5d represents the wave number *m* as a function of time along with the uncertainties in the estimates. This clarifies the fact that in general, within the uncertainties, the estimates of m generally lie between |*m*| < 100. Note that the uncertainty in the azimuthal wave number increases toward the end of the HSS when the amplitude of ULF wave power begins to decrease (Figures 2c–2e). As discussed in the previous section this is a limitation in the calculation of *m* using cross phase, as wave power decreases the uncertainty in calculating the phase difference between time series increases.

The bottom row of Figure 5 shows the distribution of *m* as a function of L; from left to right Figure 5e shows the scatter plot, 2‐D histogram and probability distribution function (PDF; chance of observing a given *y* value as a function of *x*). The scatter plot shows significant variation in *m* as a function of L; however, the 2‐D histogram and PDF show a clear preference for low *m* ULF waves, |*m*| < 20, which are larger than those reported in previous ground‐based studies (e.g., Plaschke et al., 2008) but generally consistent with previous in situ observations (Sarris et al., 2013; Tan et al., 2011). In the PDF we also see preference for *m* as a function of L. At *L* < 8 R_E_ *m* is predominantly positive and <20, between 8 and 11 R_E_ *m* is negative and ranges between −5 and −40, and at *L* > 13 R_E_ *m* is predominantly positive and <20, these regions are highlighted in the PDF by the green color where the probability is greater than 10%.

The azimuthal wave number of discrete ULF waves is dependent on the wave driver, either internally or externally driven waves, and the sign of the *m* has important implications in how ULF waves couple to energetic particles in the magnetosphere. In the subsequent sections we discuss how discrete ULF waves can couple with radiation belt electrons, the drivers of discrete ULF wave power and how they relate to the distribution of azimuthal wave numbers observed during the HSS.

## 4 The Role of Discrete Wave Power in the Magnetospheric Dynamics

Figure 6 shows the evolution of ULF wave power, period, and wave number through the inner magnetosphere, *L* = 4–8 R_E_, where ULF waves with positive azimuthal wave numbers can lead to the energization and transport of electrons leading to enhancements of radiation belt electrons (e.g., Ozeke, Mann, Turner, et al., 2014). Figure 6a shows the azimuthal component, ***B***_*ϕ*_, of ULF wave power which shows clear peaks between *L* = 6–10 R_E_ at the edge of outer radiation belt consistent with statistical ground‐based (Murphy et al., 2011; Pahud et al., 2009; Rae et al., 2012) and in situ observations (Brautigam et al., 2005; Ozeke, Mann, Murphy, et al., 2014; Takahashi & Anderson, 1992) of ULF wave power. Figure 6b shows the period, and Figures 6c and 6d show the azimuthal wave number of ULF waves within the inner magnetosphere. In this region the ULF wave periods tend to be lower than in the outer magnetosphere, between 100 and 400 s, and the azimuthal wave numbers are preferentially positive *m* < 20 (Figure 5e). The presence of discrete ULF waves, positive azimuthal wave numbers, and a peak in ULF wave power in the inner magnetosphere suggests that ULF waves may be able to couple to and energize radiation belt electrons during this HSS and geomagnetic storms via wave particles interactions.

The energization of electrons via discrete toroidal mode ULF waves in a compressed dipole can occur when the drift‐bounce resonance

(6) $$\omega -\left(m\pm 1\right)\omega_{d}=0\;$$

is satisfied, where *ω* is the wave angular frequency, *ω*_*d*_ is the bounce averaged angular drift frequency, and *m* is the azimuthal wave number. Knowing the azimuthal wave number and period of discrete ULF waves, equation (4) can be solved to determine the electron energies which will resonate with the discrete ULF wave power observed by MMS.

Though electrons can also undergo the drift‐bounce resonance with ULF wave in general only 90° pitch angles are considered in ULF wave electron energization such that electrons satisfy the drift resonance condition, and the drift‐bounce resonance is neglected (e.g., Ozeke & Mann, 2008). For simplicity, if we consider the drift resonance of 90° pitch angles then equation (5) can be solved to determine the energy of electrons which will resonate with discrete ULF waves observed during the HSS. The bounce‐averaged drift period in a magnetic dipole is given by (Walt, 1994)

(7) τd=1.557×104γβ2L1−13sinα=2πωd

where β≡vc is the particles velocity to speed of light ratio and γ≡1−β2−12. Using the substitution *T* = *KE*/*mc*^2^ where *KE* is the particles kinetic energy and *mc*^2^ is the particles rest mass, and *β* and *γ* can be rewritten as

(8) $$\beta^{2}=\frac{T\left(T+2\right)}{{\left(T+1\right)}^{2}}\;$$

(9) $$\gamma =\left(T+1\right)\;$$

Substituting equations (7)–(9) into equation (6), the energy of resonant electrons can be determined using the period and azimuthal wave number of discrete ULF waves determine from AFINO and the azimuthal wave number algorithm.

Figure 7a illustrates the energy of resonant electrons as a function of L calculated from equations (6)–(9) and using the wave periods and azimuthal wave numbers observed by the MMS spacecraft (Figure 6). Note that the drift resonance can only be solved for positive *m* values. The black points determine the resonant electron energy for only positive *m* values; however, each *m* has an associated uncertainty *δm*. The red and blue points show the resonant electron energy when the uncertainties in *m* are taken into account and *m* remains positive (e.g., *m* + *δm* > 0 or *m* − *δm* > 0). Evident in Figure 7a is that the discrete ULF wave power during this HSS would be able to resonate with electron energies throughout the radiation belt ~10s kev – ~1 MeV. Figures 7b–7e show the evolution of radiation belt electron flux from the Van Allen Probes Magnetic Electron Ion Spectrometer instrument (MagEIS, Blake et al., 2013) through the HSS. The electron energies in Figures 7b–7e correspond to the range of electron energies in Figure 7a calculated to be able to interact with radiation belt electrons. During the HSS there is clear evidence of an enhancement and energization of radiation belt electrons across the energies that may be energized by the ULF waves observed by MMS via drift resonance and during the period when ULF wave power peaks within the inner magnetosphere (cf. Figures 2c and 2d and 6a). Thus, it is possible that a portion of this energization is the result of discrete ULF wave power; we discuss this in detail in the next section.

## 5 Discussion

ULF waves are a fundamental component of inner magnetospheric dynamics especially during active geomagnetic conditions, such as geomagnetic storms (e.g., Mann et al., 2016; Murphy et al., 2015). During storms ULF wave‐particle interactions can lead to the acceleration of radiation belt electrons (e.g., Elkington et al., 2003; Ozeke, Mann, Murphy, et al., 2014; Ozeke, Mann, Turner, et al., 2014; Schulz & Lanzerotti, 1974). This acceleration is dependent on the mode structure of the wave, the azimuthal wave number, and whether the wave is discrete or broadband. For instance, discrete ULF wave power can interact with energetic particles through either the drift bounce or drift resonances, while broadband wave power can transport particles via radial diffusion. In terms of the mode structure, energization of particles via ULF waves is driven by poloidal or toroidal waves. Poloidal waves are efficient in both discrete resonances and radial diffusion due to the presence of an azimuthal electric field (e.g., Ozeke et al., 2012; Ozeke, Mann, Murphy, et al., 2014). However, toroidal waves can accelerate electrons in an asymmetric magnetic field topology (Elkington et al., 2003) typical of the compressed magnetosphere developing during the HSS studied here as a result of the interaction of the enhanced solar wind with the Earth's magnetosphere and are generally larger amplitude than poloidal waves (e.g., Murphy et al., 2011; Rae et al., 2005, 2012; Takahashi & Ukhorskiy, 2007). Hence, toroidal waves can also lead to efficient energization of electrons (Degeling et al., 2008). ULF wave power and the mode structure have both been characterized previously in case studies and statistical studies (e.g., Hartinger et al., 2013; Mathie & Mann, 2001; Murphy et al., 2011; Rae et al., 2012; Takahashi & Ukhorskiy, 2007). However, no study exists that systematically characterizes discrete ULF wave power and the azimuthal wave number of ULF waves. In this study we provide a detailed overview of the evolution of ULF waves observed by MMS on the dusk flank during a HSS leading to a moderate geomagnetic storm occurring between 24 September 2016 and 10 October 2016. This moderate geomagnetic storm resulted in a rapid enhancement in the Earth's outer radiation belt. During the storm MMS was used with two novel codes to characterize the power, structure (broadband or discrete), and azimuthal wave number of toroidal mode ULF waves.

In general, we find that ULF wave power peaks near the magnetopause and again within the inner magnetosphere (6 < *L* < 8), compare Figures 2c–2e and 6a. Near the magnetopause wave power is dominated by the compressional component, while deeper within the magnetosphere (6 < *L* < 10) the dominant wave power is a mix between toroidal and poloidal wave power (Figures 2f and 2g. This distribution of wave power and mode structure as a function of L is consistent with two generation mechanisms: (1) the solar wind driving of discrete field line resonances (e.g., Southwood, 1974) and (2) internal generation of ULF waves via unstable ion distributions (e.g., Southwood et al., 1969).

Externally driven sources of ULF wave power exist near the magnetopause, such as the KH instability (e.g., Claudepierre et al., 2008; Hasegawa et al., 2004; Rae et al., 2005) or magnetopause compressions (e.g., Murphy et al., 2016). Wave power subsequently propagates inward where it can couple to discrete field line resonances leading to a secondary peak in the toroidal and poloidal components of ULF wave power (e.g., Southwood, 1974). Conversely, localized poloidal ULF wave power in the inner magnetosphere can be driven via unstable ion distributions, typically observed during the main phase of geomagnetic storms and ring current enhancements (e.g., Takahashi et al., 1987). Internal ion‐driven poloidal mode waves typically have negative azimuthal wave numbers, whereas externally driven waves propagate antisunward, that is, have positive azimuthal wave numbers on the dusk flank. Utilizing MMS, AFINO, and a new algorithm to calculate *m*, we can investigate the evolution of toroidal mode ULF wave periods and azimuthal wave numbers throughout the HSS.

Throughout the HSS we find that discrete toroidal power is frequently present over a wide range of L, with typical periods in the 100‐ to 400‐s range with shorter periods typically observed at lower *L* values (Figure 3). This distribution of discrete toroidal ULF wave power is consistent with the magnetospheric Alfven continuum, whereby the period of discrete ULF waves is longest near the magnetopause and decreases moving inward where there is a sudden increase in wave period at the plasmapause, a result of the increase in magnetospheric mass density (e.g., Ozeke & Mann, 2008). However, in our results there is no evidence for the jump in wave period at the plasmapause. This may be a result of the azimuthal wave number of the ULF waves or the magnetospheric density profile. Lee (1996) demonstrated that the relative jump in frequency of ULF waves around the plasmapause boundary decreases as azimuthal wave number increases; the variation in azimuthal wave numbers observed here may account for the lack of any clear jump in wave period. In contrast there may be no clear jump in frequency as there is no clear plasmapause boundary, the result of either the erosion of the plasmasphere (e.g., Carpenter & Anderson, 1992; Goldstein et al., 2014) or formation of a plasmasphere boundary layer (Liu et al., 2013) which can occur during geomagnetic storms such as the HSS and storm studied here.

In general, although there are sustained intervals of discrete and broadband power during this storm, there is a mix of power throughout this event with no clear correlations with other solar wind parameters (Figures 1 and 5). Theory (A. Hasegawa, 1975), simulations (Claudepierre et al., 2008), and observations (Rae et al., 2005) have all shown that discrete ULF wave power can develop during periods of high‐speed solar wind as a result of KH activity along the magnetopause flanks. However, while there are periods of sustained high‐speed solar wind throughout the HSS, there are also periods of large fluctuations in solar wind density, dynamic pressure, and IMF Bz (Figure 1). These solar wind‐driven processes can also drive ULF waves in the inner magnetosphere, directly through buffeting of the magnetopause via fluctuation in solar wind density and dynamic pressure (Kepko et al., 2002; Murphy et al., 2015) or indirectly by substorms and particle injections resulting from periods of southward IMF (e.g., Takahashi et al., 1987). The mix of solar wind conditions observed during the HSS suggests that discrete ULF wave power is driven both externally and internally during this HSS interval.

Using the unique configuration of MMS, we estimate the value of *m* for each discrete toroidal mode interval using the cross‐phase technique described in section 2.3 (Figure 4) to further investigate the driver of discrete toroidal ULF wave power during the HSS. This approach has the crucial advantage of removing any phase‐wrapping ambiguity in estimates of *m*. During discrete wave events the azimuthal number varies significantly, with |*m*| < 100 (Figures 5c and 5d). These *m* values for the toroidal mode ULF wave are generally consistent with previous ground‐based (Plaschke et al., 2008) and in situ studies (Sarris et al., 2013; Tan et al., 2011). During the HSS, near the magnetopause (*L* > 13 R_E_) and in the inner magnetosphere (*L* < 8 R_E_) the azimuthal wave number is predominantly positive, while between these regions (8 < *L* < 13 R_E_) is predominantly negative, as shown in Figure 5e. The observed periods and azimuthal wave numbers are consistent with both internal and external drivers of ULF wave power as described above. ULF waves at *L* > 13 and 6 < *L* < 8 R_E_ nominally externally driven and those between 8 and 13 R_E_ being predominantly internally driven.

In a statistical study of Pc3–5 waves observed by Active Magnetospheric Particle Tracer Explorers/Charge Composition Explore Anderson et al. (1990) found that the occurrence of Pc5 wave power on the dusk flank peaked between 7 and 9 R_E_. These waves typically have moderate to large (Takahashi et al., 1985) and negative (Lin et al., 1988) azimuthal wave numbers and have been suggested to be driven internally by unstable ion distributions developing during storms and substorms (e.g., Ozeke & Mann, 2001). The discrete ULF waves observed during the HSS between 8–13 R_E_ on the dusk flank show the same characteristics as internally driven waves and thus are likely the result of unstable ion injections driven by either substorm injections or an enhanced ring current ion population during the HSS.

Near the magnetopause (*L* > 14 R_E_) and in the inner magnetosphere (6–8 R_E_) the discrete ULF wave power observed during the HSS is consistent with that developing as a result of the KH instability. In both regions wave power peaks (Figure 2) and the azimuthal wave number is predominantly positive and less than 20 (Figure 5e). Further, at the magnetopause boundary the wave power is predominantly compressional and a mix between toroidal and poloidal in the inner magnetosphere (Figure 2). This distribution of wave amplitudes, mode structure, and azimuthal wave number is consistent with the generation of discrete wave power via the KH instability which couples locally to field line resonances within the inner magnetosphere and propagates antisunward (positive m; Southwood et al., 1969).

In relation to wave‐particle interactions and radiation belt electron dynamics, the presence of discrete toroidal ULF wave power, with preferred positive *m* values in the inner magnetosphere and clear enhancement of the outer radiation belt, suggests that discrete toroidal ULF waves may be a key component to radiation belt dynamics. Discrete toroidal ULF waves can interact with and energize electrons when they satisfy the drift bounce and bounce resonant conditions. Generally, radiation belt studies analyzing electron acceleration via ULF waves neglect the drift‐bounce resonance and only consider the drift resonance. This is because the resonant energy of electrons that satisfy the drift‐bounce resonance is typically too low or too high to be considered in radiation belt dynamics (Ozeke & Mann, 2008). Considering only 90° pitch angle electrons and neglecting the drift‐bounce resonance, we can solve the drift resonance to determine the energy of electrons that discrete ULF waves observed during the HSS can interact with. Figure 7a shows the energy of resonant electrons as a function of L for the discrete toroidal ULF waves and azimuthal wave numbers observed. From this it is clear that discrete toroidal ULF wave power can accelerate electrons throughout the radiation belt from ~10s of keV to ~MeV energies. During this HSS and moderate geomagnetic storm a clear energization of the radiation belt across these energies is observed by the Van Allen Probes (Figures 7b–7e). This is consistent with discrete toroidal ULF wave power being a potential component in radiation belt dynamics during geomagnetic storms. These results have important implications as the energization time scales for discrete wave‐particle interactions are faster than the time scales of stochastic broadband processes such as radial diffusion (e.g., Degeling et al., 2008; Elkington et al., 2003). Future work statistically characterizing the distribution of discrete versus broadband ULF wave power and azimuthal wave numbers of both the toroidal and poloidal modes during storms will help to develop a full understanding of ULF waves within the magnetosphere and diagnosis the driver of these waves. This will also allow the assessment of the relative importance of discrete (Degeling et al., 2013) and broadband ULF wave power (e.g., Ozeke, Mann, Turner, et al., 2014) using radiation belt models to fully understand the overall dynamics of the Earth's outer radiation belt.

## 6 Conclusions

In this paper we have presented a detailed study characterizing ULF wave power, mode structure, and azimuthal wave number during a moderate geomagnetic storm driven by a HSS. Using the unique configuration of the multispacecraft MMS mission and two novel algorithms, we systematically identify periods of discrete ULF wave power during the storm and determine the characteristics of these wave periods and azimuthal wave numbers. The AFINO code (Inglis et al., 2015, 2016) uses a data‐model comparison to identify intervals of discrete ULF wave power from observations of the magnetic field from MMS. During these identified intervals, a new cross‐phase technique is used to identify the phase difference of ULF waves between the four MMS spacecraft. A linear regression of the spacecraft separation and cross phase are then used to calculate the azimuthal wave number. This new technique coupled with observations from MMS has two benefits to previous techniques; first, the linear regression provides an estimate of the uncertainty in the azimuthal wave number and second, the close separation of the MMS spacecraft and multipoint observations allows phase differences to be unambiguously determined in the calculation *m* (hence, phase wrapping can be ignored). The results presented here demonstrate the utility of the MMS mission and the AFINO and cross‐phase codes to systematically characterize period of broadband and discrete of ULF waves and the corresponding azimuthal wave number during discrete wave events. More importantly, these results allow the generation mechanism of ULF waves in the inner magnetosphere to be determined as well as the energy of electrons which discrete ULF waves can couple to via wave‐particle interactions.

In general, we find that ULF wave power tends to be compressional at high L and a mix between toroidal and poloidal modes at lower L. Wave power peaks near the magnetopause (*L* > 10) and shows evidence of a secondary peak in the inner magnetosphere (6 < *L* < 8). Throughout the geomagnetic storm there are prolonged periods of both discrete and broadband power; however, no clear solar wind driver for either wave type is observed. The period of discrete ULF waves generally lies in the Pc5 wave band (100–400 s), and the toroidal azimuthal wave number is typically low but is generally positive at higher *L* values (*L* > 13 R_E_) and in the inner magnetosphere (6 < *L* < 8 R_E_) and negative between these regions (8 < *L* < 13 R_E_). At high and low *L* values these wave periods and azimuthal wave numbers are consistent with external driving of ULF waves via the KH instability coupling to local field line resonances in the inner magnetosphere. At intermediate *L* values (8 < *L* < 13 R_E_), the observed ULF waves are consistent with internal generation via unstable ion distributions.

Using the calculated period and azimuthal wave numbers of discrete toroidal mode ULF waves, we are further able to calculate the expected resonance condition of discrete toroidal ULF waves with radiation belt electrons. We find that discrete toroidal ULF wave power can couple to a significant portion of the radiation belt electron population, ranging from ~10s of keV – 1 MeV, energies at which the Van Allen Probes show a clear enhancement of the radiation belt population.

Overall, this work demonstrates that storm time discrete ULF waves are the result of both external and internal generation, via the KH instability and Field Line Resonances and unstable ion distributions (respectively). More importantly, this work clearly demonstrates that discrete toroidal ULF waves can couple to the core radiation belt electron population and may be an important factor in storm time electron dynamics which are not currently considered in radiation belt models. Future work is needed to investigate the azimuthal wave number of poloidal mode ULF waves as well statistically characterize the distribution of discrete and broadband ULF wave power and azimuthal wave numbers of both the toroidal and poloidal modes during geomagnetic storms. This would allow the generation mechanism of ULF waves to be characterized as a function of storm phase and storm driver and would allow the spatial and temporal distribution of the azimuthal wave number of ULF waves to be investigated. In general, the azimuthal wave number of ULF waves is the most poorly prescribed parameter in radiation belt models. Such a study would help to assess the overall importance of ULF waves in inner magnetosphere and radiation belt dynamics.
